# Processing and Properties of High-Entropy Ultra-High Temperature Carbides

**DOI:** 10.1038/s41598-018-26827-1

**Published:** 2018-06-05

**Authors:** Elinor Castle, Tamás Csanádi, Salvatore Grasso, Ján Dusza, Michael Reece

**Affiliations:** 1 Queen Mary, University of London, Mile End Road, London, E1 4NS UK; 2 Institute of Materials Research, Slovak Academy of Sciences, Watsonova 47, 04001 Kosice, Slovakia

## Abstract

Bulk equiatomic (Hf-Ta-Zr-Ti)C and (Hf-Ta-Zr-Nb)C high entropy Ultra-High Temperature Ceramic (UHTC) carbide compositions were fabricated by ball milling and Spark Plasma Sintering (SPS). It was found that the lattice parameter mismatch of the component monocarbides is a key factor for predicting single phase solid solution formation. The processing route was further optimised for the (Hf-Ta-Zr-Nb)C composition to produce a high purity, single phase, homogeneous, bulk high entropy material (99% density); revealing a vast new compositional space for the exploration of new UHTCs. One sample was observed to chemically decompose; indicating the presence of a miscibility gap. While this suggests the system is not thermodynamically stable to room temperature, it does reveal further potential for the development of new *in situ* formed UHTC nanocomposites. The optimised material was subjected to nanoindentation testing and directly compared to the constituent mono/binary carbides, revealing a significantly enhanced hardness (36.1 ± 1.6 GPa,) compared to the hardest monocarbide (HfC, 31.5 ± 1.3 GPa) and the binary (Hf-Ta)C (32.9 ± 1.8 GPa).

## Introduction

Ultra-High Temperature Ceramics (UHTC’s) are a limited and select set of carbides, nitrides and borides of the group IV and V transition metals, which are typically defined as having melting temperatures >3300 K; with HfC exhibiting the highest known melting point of all materials (4232 ± 84 K)^1^. UHTC’s also display high hardness, elastic modulus and resistance to thermal shock and chemical attack. For technologies such as high temperature nuclear reactors, jet engines and hypersonic vehicles they therefore represent the only suitable class of materials available to make or protect components that are placed under the most extreme of operating environments. As these developing technologies become more advanced and more demanding, UHTC’s are coming under increasing pressure to perform under more diverse operating conditions. A greater selection of UHTC’s that exhibit a much broader range and combination of physical, chemical and mechanical properties are therefore required to meet these demands.

Towards this, some inspiration has recently been taken from the field of metallurgy; in which the useful compositional space for the exploration of new alloys was drastically increased upon the discovery of High Entropy Alloys (HEAs)^2,3^. These materials consist of single-phase crystalline solid solutions (crystallographic order), comprised of a random arrangement of >4 metallic components in near-equiatomic proportions (chemical disorder). Such compositions were previously expected to produce multiphase alloys, consisting of brittle intermetallics; and were therefore expected to be impractical for most applications. In reality, it was discovered that single-phase materials could be stabilised via their enhanced molar configurational entropy (ΔS_*mix*_ = *R*ln*N*, where *N* is the number of equimolar components and *R* is the gas constant)^4,5^, which minimises the Gibb’s free energy (*G* = *H* − *TS*, where *H* is enthalpy, *S* is entropy, and *T* is temperature) by boosting the *–TS* term. The *–TS* term becomes more dominant at elevated temperatures, suggesting that high entropy phases could be particularly stable at high temperatures.

Applying the same entropy-stabilisation concept to UHTC’s, a number of multicomponent diborides were recently fabricated^6^ and investigated using aberration-corrected scanning transmission electron microscopy (AC STEM), with high-angle annular dark-field and annular bright-field (HAADF and ABF) imaging and nanoscale compositional mapping. The analysis indicated that, for the majority of the compositions studied, the metal components displayed no discernible ordering or segregation between the atomic layers of the metallic sublattice; indicating that high entropy materials, with random metal site occupancy, had successfully been fabricated. This therefore opens up a wide new range of potential UHTC compositions to explore, and has subsequently generated much interest in high entropy ceramics within the UHTC community. An initial assessment of the hardness and oxidation properties of the high entropy diborides suggests that these properties are generally improved above and beyond their component mono-diborides and above that predicted by a rule of mixtures approximation. In HEA’s, this relative enhancement in key materials properties is attributed to the chemical and structural disorder inherent in the high entropy phase. The incorporation of atoms with different atomic sizes, crystal structures and bonding preferences into the same crystal lattice can lead to local lattice distortions (localised strains) and fluctuations in the local chemical environment; which would be expected to have an influence on the movement of diffusing species, defects and dislocations^7^. Thus, the observed ‘abnormal’ mechanical and oxidation behaviour in the high entropy diborides indicates that similar phenomena may also apply to high entropy ceramics.

As well as the diborides, “multi-principle component carbides” based on 5 or more near-equiatomic transition metal elements have also been successfully fabricated in the form of coatings. (Ti-Zr-Nb-Ta-Hf)C (with either Ti or Ta exchanged for Si) have been fabricated as biocompatible coatings for metal implants^8^ and (Ti-Zr-Hf-V-Nb-Ta)C coatings with improved tribological behaviour have also been produced^9^. The (Ti-Zr-Hf-V-Nb-Ta)C coating exhibited an elastic modulus of 337 GPa, similar to that of the monocarbides; yet an extremely high hardness of 43–48 GPa, which is markedly higher than any of the component monocarbides.

To date, the successful synthesis of a bulk high entropy carbide has not been reported. In the present work, we investigated the synthesis of bulk UHTC high entropy carbides, and performed an initial examination of their micromechanical behaviour.

## Results and Discussion

### Solid solution formation

The five UHTC monocarbides, with T_m_ > 3273 K, are HfC, TaC, ZrC, NbC and TiC. All five of these stoichiometric carbides exhibit a cubic rock salt crystal structure (space group Fm-3m, No. 225) at room temperature; and many of the mixed pseudo-binary (e.g. HfC-TaC) carbide systems investigated between these five carbides form single phase solid solutions^10–12^. In addition, the five transition metals have similar atomic radii, so between them satisfy the Hume-Rothery-based atomic size misfit parameter, *δ* ≤ 6.6%^3^, and hence many HEA compositions have been fabricated based on these elements^13–17^.

Two target compositions based on these five UHTC monocarbides were chosen to investigate: (Hf-Ta-Zr-Ti)C and (Hf-Ta-Zr-Nb)C. Compositions involving only four metallic components were selected in order to simplify the systems so that the composition which was found to be the most stable could be used as a starting point for the systematic addition of further metallic elements in future work. High purity precursor powders were selected, and the compositions ball milled and sintered by Spark Plasma Sintering (SPS), as described in more detail in the materials and methods section.

**Figure 1:**
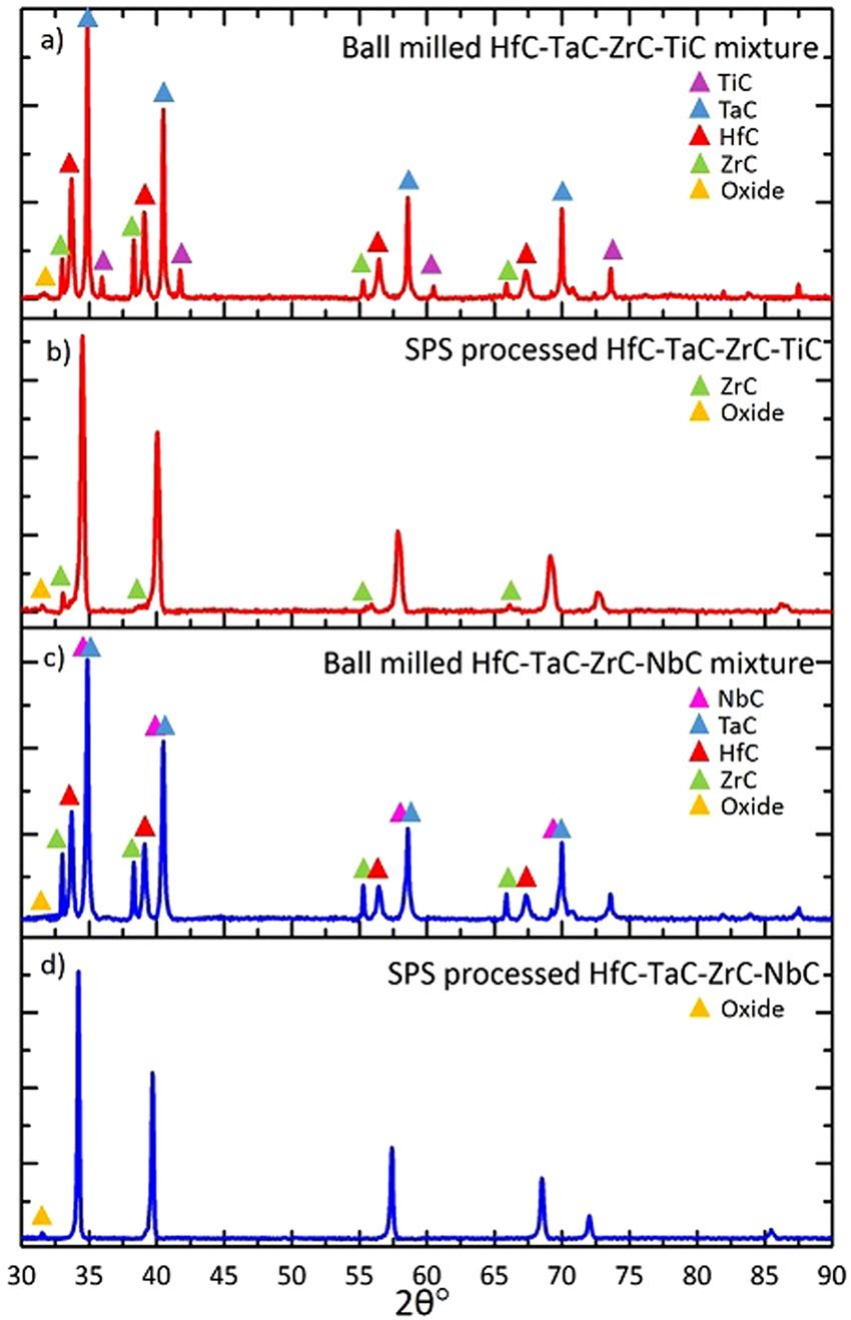
XRD data taken from: (**a**) the ball milled HfC/TaC/ZrC/TiC mixture; (**b**) the (Hf-Ta-Zr-Ti)C sample after sintering by SPS; (**c**) the ball milled HfC/TaC/ZrC/NbC mixture; and (**d**) the (Hf-Ta-Zr-Nb)C sample after sintering by SPS.

The XRD patterns of the ball milled mixtures and SPS sintered samples are shown in Fig. 1. A relative density of 94% was achieved in both SPS sintered samples. For the HfC-TaC-ZrC-TiC composition, the majority of the XRD peaks relating to the individual carbide powders have disappeared to leave one main set of rock salt structure (FCC) peaks following sintering; indicating that there was significant inter-diffusion between the different elements and that the formation of a multi-metal carbide phase occurred. However, small ZrC peaks remain evident in the XRD pattern; relating to the incomplete diffusion of ZrC. A small amount of oxide phase is also present. In the SPS sintered HfC-TaC-ZrC-NbC sample there is only one set of peaks evident in the XRD pattern, except for a small amount of oxide phase, suggesting that there has been complete inter-diffusion of all elements to produce a single, multi-metal FCC carbide phase.

Since there was full inter-diffusion of the ZrC in the (Hf-Ta-Zr-Nb)C composition, this raised the question of why the ZrC finds it easier to diffuse into the mixed carbide phase when the TiC is replaced with NbC. It is not a trivial task to determine the diffusion processes occurring among multiple inter-diffusing components in a crystal structure involving two sublattices (C and metal); and hence we have extrapolated from what we learnt from the current literature on inter-diffusion in rock-salt structured monocarbides. It is generally accepted that metal self-diffusion is independent of C self-diffusion in the carbides; since C self-diffusion rates have been found (in NbC, ZrC, TiC and V_6_C_5_) to be dependent upon C concentration, whereas metal self-diffusion has been found to be independent of C concentration^18–21^. The C self-diffusion process is thought to be a two-step process, in which the C atom can occupy an empty tetrahedral site before migrating into a nearest neighbour C-vacancy. Therefore, for C diffusion, only the energy associated with atomic migration is necessary for the process to occur. By contrast, metal self-diffusion can only occur upon the formation of a nearest neighbour metal vacancy; and hence depends on both the vacancy formation energy and the energy for migration. As such, measured C self-diffusion coefficients tend to be several orders of magnitude higher than metal self-diffusion coefficients in carbides^22^.

Based on this, we determined that C self-diffusion in the mixed carbides is not likely to be the rate-limiting process, and therefore the self- and inter-diffusion rates of the component metals would be the main limiting factors for the formation of a fully mixed high entropy carbide phase. In this case, the rate of diffusion will depend upon both the host and the diffusing species. As mentioned above, in the case of metal self-diffusion, the formation of a nearest neighbour vacancy is a necessity. In this case the energy required for vacancy formation is an important, and potentially dominating, contribution to metal self- (and inter-) diffusion in the carbides. Table 1 contains values for the metal vacancy formation energies in the carbides, calculated by Yu *et al*. using electronic structure density functional theory^23^. This shows that Ta has the lowest vacancy formation energy of all of the metals in their respective carbides, closely followed by Nb. In contrast, the vacancy formation energies of Ti, Hf and Zr are 2–3 times higher.

**Table 1:** Physical parameters of the refractory metal elements (and their carbides) employed in the two attempted high entropy carbide compositions.

Element	Atomic Radius (Å)^a^	Lattice parameter of stoichiometric carbide (Å)	Melting temperature of stoichiometric carbide (°C)	Calculated metal vacancy formation energies in the carbides (eV)^k^
Hf	1.564	4.637^b^	3959^g^	9.3
Ta	1.430	4.452^c^	3768^g^	3.5
Zr	1.590	4.692^d^	3427^h^	9.4
Nb	1.448	4.470^e^	3600^i^	4.1
Ti	1.429	4.326^f^	3027^j^	8.6

^a^Fluck *et al*.^46^, ^b^Lengauer *et al*.^47^, ^c^Bittner *et al*.^48^, ^d^Chase, Juenke^49^, ^e^Wong-Ng *et al*.^50^, ^f^Capkova *et al*.^51^, ^g^Cedillos-Barazza *et al*.^1^, ^h^Fernández Guillermet A.^52^, ^i^Smith *et al*.^53^, ^j^Frisk, Karin^54^, ^k^Yu *et al*.^23^.

In the case of the inter-diffusion of metals between different carbides, a handful of diffusion couple studies have been performed between TaC, HfC and ZrC^10^ and between HfC and TiC^24^. From these experiments it was determined that TaC tends to act as the host material, with Hf and Zr inter-diffusion into TaC occurring faster than Ta inter-diffusion into the HfC or ZrC. This is likely to be due to the lower vacancy formation energy of Ta, making the TaC more likely to accommodate the diffusing Zr or Hf species. In the same way, the lower relative rates of diffusion of Ta into HfC or ZrC is likely to be due to the higher vacancy formation energies of Hf and Zr, and not due to the diffusivity of the Ta species itself. It therefore appears that TaC is a useful component to employ in high entropy carbide compositions in order to encourage better inter-diffusion of all component species.

The inter-diffusion coefficients of Hf and Zr into TaC were measured to be of a similar order of magnitude (0.35 × 10^−12^ m^2^s^−1^ and 1.7 × 10^−12^ m^2^s^−1^, respectively, at 2000 °C) while in the HfC-ZrC diffusion couple, both metals displayed equal inter-diffusion rates^10^. In the HfC-TiC diffusion couple, Ti was found to diffuse faster into HfC than Hf into TiC. This, and the fact that TiC exhibits the lowest melting temperature (highest atomic mobility at a given temperature) and smallest lattice parameter (and smallest atomic size of the metallic element) of the five carbides (Table 1) suggests that TiC is likely to be the fastest diffusing species, in spite of its relatively high vacancy formation energy. This is supported by the present observation of the complete loss of a distinct TiC peak in both high entropy carbide compositions.

Using the above inter-diffusion analysis and arguments, vacancy formation energies, known melting points (relative atomic mobility), metal atomic radius and lattice parameters of the individual carbides (Table 1), the following order for the expected slowest to fastest inter-diffusing metal species in the carbides may be suggested: Ta < Zr ~ Hf < Nb < Ti. In spite of its intermediate lattice parameter, metal atomic radius and melting point, Ta has been placed as the slowest inter-diffusing species due to its observed tendency to act as the host material and slow relative diffusion rates into the other carbide components. While Hf and Zr appear to have similar inter-diffusion coefficients based on the literature, Zr is suggested to be the slower of the two species based on its slightly higher vacancy formation energy, larger carbide lattice parameter and the experimental observation in this work of incomplete mixing of the ZrC in the HfC-TaC-ZrC-TiC composition. Given the markedly lower melting point of the ZrC (higher expected atomic mobility) in comparison to the HfC, and assuming that its vacancy formation energy is constant (i.e. independent of the starting powder composition), this suggests that the lattice parameter mismatch of the component monocarbides may be a key determining/predictive factor in the ability to form solid solutions; analogous to the use of the Hume-Rothery-based atomic size mismatch parameter. The NbC species was the most difficult to place in this order. Since the lattice parameter, melting temperature and atomic radius values of Nb all lie in the middle of the five carbide components NbC < TiC is likely. Drawing upon its lower vacancy formation energy and the suggestion that carbide lattice parameter mismatch is the most important determining factor for inter-diffusion ability, Nb has then been tentatively placed as a faster diffusing species than Ta, Zr and Hf.

**Figure 2:**
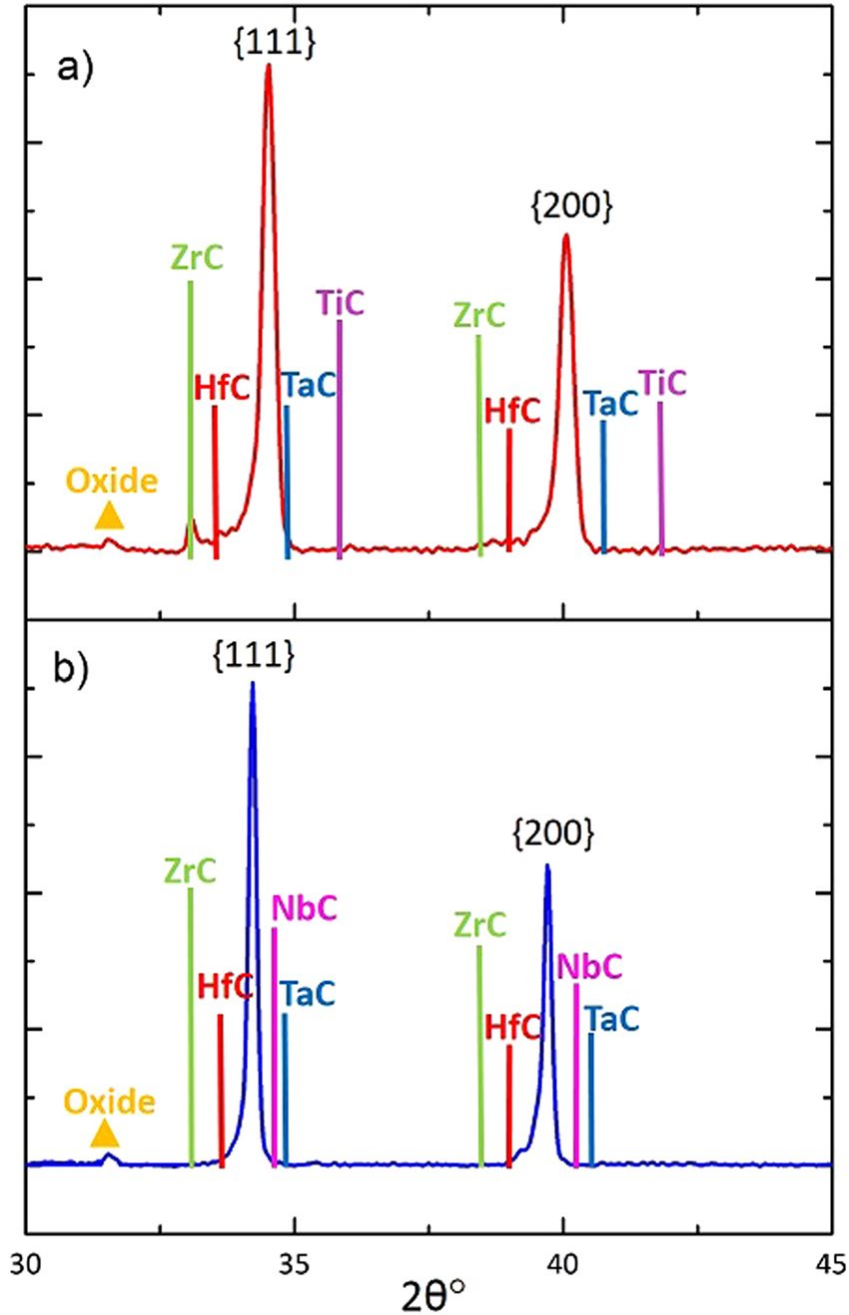
Close-up of the first two ({111} and {200}) XRD peaks with expected peak positions for the component monocarbides for: (**a**) the sintered (Hf-Ta-Zr-Ti)C sample; and (**b**) the sintered (Hf-Ta-Zr-Nb)C sample.

To investigate further, Fig. 2 shows a closer inspection of the first two ({111} and {200}) XRD peaks for the two multicomponent compositions studied, with the peak sticks for the component monocarbide diffraction patterns superimposed to show clearly their expected relative peak positions. In Fig. 2(a) it can be seen that the TiC diffraction peaks are at much higher 2θ° values than the rest of the monocarbides due its much smaller lattice parameter. The absence of a peak here demonstrates that the TiC had fully diffused. Since the TiC is expected to be the fastest inter-diffusing component, it is logical to suggest that this became fully incorporated into the TaC ‘host’ first. This would have the effect of initially reducing the lattice parameter of the mixed/high entropy carbide phase; in turn, making it more difficult for the larger Zr to inter-diffuse into the mixed carbide phase and slowing down the kinetics of the ZrC inter-diffusion. By contrast, in Fig. 2(b) it can be seen that the NbC peaks are at lower 2θ° values (larger lattice parameter) than those of TaC; and would therefore have the effect of increasing the lattice parameter of the TaC upon inter-diffusion, making it easier and more kinetically favourable for the ZrC to be incorporated into the high entropy carbide phase. This may therefore explain the observed differences in the ability of the ZrC to completely diffuse into the mixed carbide phase under the same processing conditions, as a function of the starting powder composition. Indeed, it can be seen that the XRD peaks are shifted to higher 2θ° values (smaller d-spacings) in the (Hf-Ta-Zr-Ti)C phase in comparison to the (Hf-Ta-Zr-Nb)C peaks. While the kinetics of high entropy carbide formation are slower in the HfC-TaC-ZrC-TiC composition, the decrease in intensity of the ZrC peak shows that inter-diffusion is occurring and it is therefore likely that with much longer processing times it would be possible to produce a single phase high entropy carbide of this composition.

Closer inspection of the first two XRD peaks in both materials (Fig. 2) also reveals more of the detail in the asymmetry of the peaks, which are skewed towards lower 2θ° values. This is related to the incomplete diffusion of the ZrC and potentially the HfC too; which seems likely given their reported similarity in diffusion behaviour. There is a peak in the expected ZrC position; indicating that some of the ZrC is present in its pure monocarbide form; but this is not the case for the HfC. The asymmetric skew of the peaks suggests that some incomplete, intermediate solid solutions are forming with a range of lattice parameters dependent on their ZrC and HfC concentration. This peak asymmetry also appears to a lesser extent in the ‘single-phase’ (Hf-Ta-Zr-Nb)C composition; suggesting that, to a lesser extent, the chemical mixing may not be fully homogeneous within this sample.

### Process optimisation

Since the (Hf-Ta-Zr-Nb)C composition more readily forms a single phase solid solution than the (Hf-Ta-Zr-Ti)C composition, this composition was selected for further characterisation and processing optimisation. The sample which was initially produced using a 2 min dwell at the highest sintering temperature (2300 °C) had a density of 94% and displayed some chemical inhomogeneity when investigated by EDS. While in the majority of the sample all four metallic elements could be detected, there also existed regions in which only Hf and Ta were detected in the chemical analysis. Coincidentally, a solid solution between HfC and TaC would give a lattice parameter similar to the main high entropy solid solution and hence its presence in the material would be difficult to identify from the XRD pattern. On increasing the dwell time to 5 min, the density was increased to 97%, however, chemical inhomogeneity was still evident in the EDS analysis. The reader is referred to Supplementary Fig. S1 for the corresponding SEM and EDS analysis.

**Figure 3:**
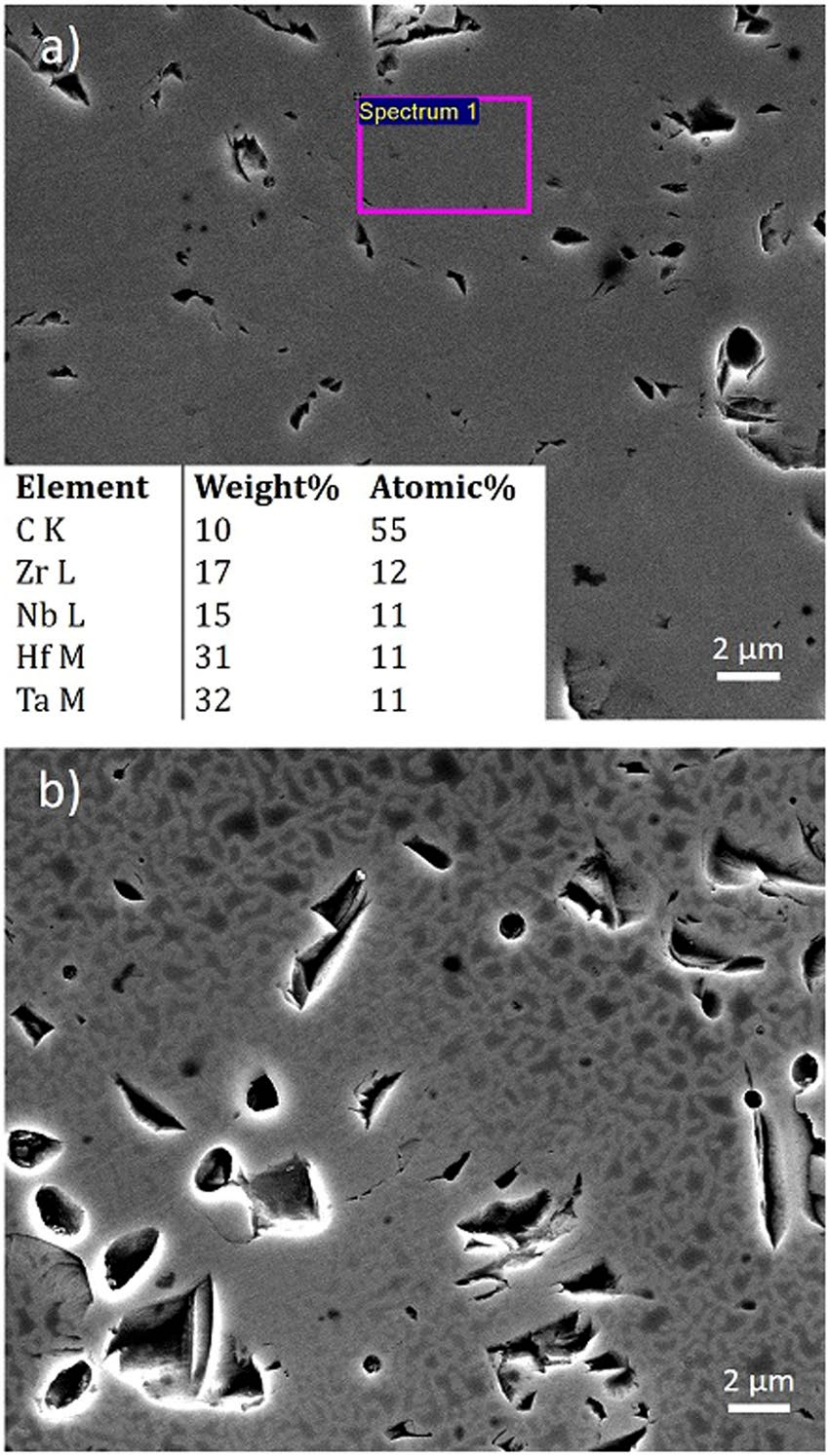
SEM images of the (Hf-Ta-Zr-Nb)C sample which was sintered for 10 min at 2300 °C, showing: (**a**) chemical homogeneity in the EDS analysis at the edge of the sample; (**b**) some form of chemical decomposition in the centre of the sample.

The dwell time at 2300 °C was then increased to 10 min, leading to a further increase in density to 98%. In this case (Fig. 3(a)) the edge of the sample appeared to be fully chemically mixed with the target composition. However, in the centre of the sample (Fig. 3(b)) the contrast in the SEM image showed that some form of chemical decomposition had taken place. This occurred on a relatively fine scale, hence the interaction volume associated with EDS analysis was too large to target the individual regions and examine which elements were separating out of the chemically mixed structure. The corresponding XRD pattern is given in the supplementary information (Supplementary Fig. S2) and appears to show a single set of FCC rock-salt phase peaks; hence the two phases which have separated must both be of the cubic rock-salt structure with lattice parameters that are similar in magnitude, leading to an overlap in their peak positions. Since it appears that the centre of the sample cools slower than the edge of the sample, it is likely that these slower cooling rates have allowed the phase separation to occur in the centre of the sample; indicating the presence of a miscibility gap in the (Hf-Ta-Zr-Nb)C system. In the pseudo-binary carbide phase diagrams, miscibility gaps are often predicted to be present^25–28^ and in some cases have been experimentally observed^29–32^. In these studies, the deliberate decomposition of binary carbide and nitride samples was performed in order to produce materials with enhanced hardness. A precipitation hardening effect was observed due to the *in-situ* production of a crystallographically coherent dual-phase microstructure. To produce these microstructures, the compositions typically had to be held at the highest temperature possible within the immiscible region (typically 1300 °C) for up to 500 h. By contrast, decomposition in the (Hf-Ta-Zr-Nb)C sample in the present investigation appears to occur over a much shorter timescale (around 20–30 min), suggesting that the high entropy phase is not thermodynamically stable down to room temperature and that the miscibility gap exists at high enough temperatures enabling accelerated chemical separation due to more rapid diffusion. While this may not favour the formation of a stable, single high entropy phase; it may offer other advantages in terms of the potential to rapidly produce *in situ* formed nanocomposite refractory ceramics. To increase the stability of the high entropy phase, more metallic components can be introduced to the base (Hf-Ta-Zr-Nb)C composition investigated in order to boost the entropy-stabilisation effect.

Since three samples were made under different hold times at the same nominal temperature (2 min, 5 min and 10 min at 2300 °C), in theory they should have experienced the same cooling conditions (from 2300 °C to room temperature in 20–30 min). This raises the question of why the sample held for 10 min at 2300 °C was the only sample to undergo chemical decomposition. One potential explanation could be that the high entropy phase exhibits a low thermal conductivity. Hence, the more the high entropy phase forms, the longer it takes for the centre of the sample to reach the set sintering temperature, the more likely it is to overheat due to the reduced ability for the heat to diffuse away, and the longer the sample will take to cool. This would mean that only the 10 min dwell time was enough to attain the target temperature (and possibly overheat) in the centre of the sample, allowing complete chemical mixing and production of the high entropy phase; and therefore lower the thermal conductivity of the samples, reduce heat flow out of the centre and hence further reduce the cooling rate in the centre of this sample in comparison to the 2 and 5 min dwell time samples. Note that the thermal conductivity would be expected to become even lower upon this decomposition into a two phase structure, hence it may be a self-perpetuating process. Further modelling studies and thermal conductivity measurements will be needed in order to test this theory and understand the miscibility behaviour.

Since the sample sintered for 5 min at 2300 °C was chemically inhomogeneous and the sample sintered for 10 min at 2300 °C was decomposed, a 7 min dwell time was tested. This was done in combination with an additional pressureless sintering step, in which the powder was heated under pressureless sintering conditions to 1800 °C and held for 10 min. This step ensured that any gasses produced from low temperature oxides, chlorine impurities in the starting powder (present due to the cracking process used in the extraction of the carbides from their minerals) and from the stearic acid, which was used as a process control agent in the ball milling process, were able to escape the sample prior to sintering. The removal of trapped gas pores from the microstructure resulted in an increase in density to 99%. The reader is referred to Supplementary Fig. S3 to see the effect of this step on the microstructure.

The optimised sample which was degassed and sintered for 7 min at 2300 °C was analysed by EDS. It was found that in all the analysed regions, between the edge and the centre of the sample, the target composition was achieved, with no chemical inhomogeneity and no detectable impurities or oxygen present (within the limits of the EDS analysis). The corresponding XRD pattern (see Supplementary Fig. S4**)** confirms that a single phase material was produced and that there was no crystalline oxide phase present, as a result of the degassing step. A high purity, dense and homogeneous high entropy carbide has therefore been successfully produced.

### Nanoindentation hardness and modulus

In order to compare the micromechanical properties of the (Hf-Ta-Zr-Nb)C high entropy carbide to the base monocarbides, HfC and TaC samples were also produced by ball milling and SPS sintering involving a 2 min dwell time at 2300 °C. This led to densities of >96% in both the HfC and TaC samples. The other base ZrC and NbC monocarbides that are considered were fabricated using a similar SPS sintering route (powders from H.C. Starck, sintering at 2200 °C under 35 MPa for 30 min) in previous work involving the same authors^33^ and hence they were subjected to an identical nanoindentation testing regime to that applied in the present work. Therefore, it is considered that any difference in mechanical behaviour observed between the four monocarbides (HfC, TaC, ZrC, NbC) derives only from their intrinsically different physical properties. Additionally, a (Hf-Ta)C sample was also fabricated in the present study, to facilitate a comparison between a relevant binary solid solution and the optimised high entropy carbide, using the route optimised for the high entropy carbide composition (degassing and sintering for 7 min at 2300 °C), resulting in a sample that was also >96% dense. The above mentioned samples were then subjected to nanoindentation measurements of hardness and modulus.

Inspection of the indents by SEM (see examples of the HfC and high entropy carbide samples in Supplementary Fig. S5) revealed that all four of the tested materials display similar features. The microstructure and indents on the ZrC and NbC samples can be found in the paper by Balko *et al*.^33^. The majority of the indents did not exhibit cracks on their walls. In some places cracks were observed at the periphery of the indents, which indicates that their generation only occurs at higher loads/depths. The morphology of the indents was concave or regular triangular-shape, which suggests that no pile-up had occurred around them. This is in agreement with the known brittle nature of the transition metal carbides (TaC, HfC), with limited plasticity, and therefore indicates a similar deformation behaviour in the binary ((Hf-Ta)C) and high-entropy transition metal carbides ((Hf-Ta-Zr-Nb)C). Based on the observed sink-in behaviour (i.e. no pile up of material around the indents) of the indents, it was confirmed that the measured nanoindentation data were correctly evaluated by the Oliver-Pharr method^34^. Any pile-up of material would lead to a greater surface area in contact with the indenter in comparison to that assumed by the model, which would subsequently overestimate the hardness and modulus.

**Figure 4:**
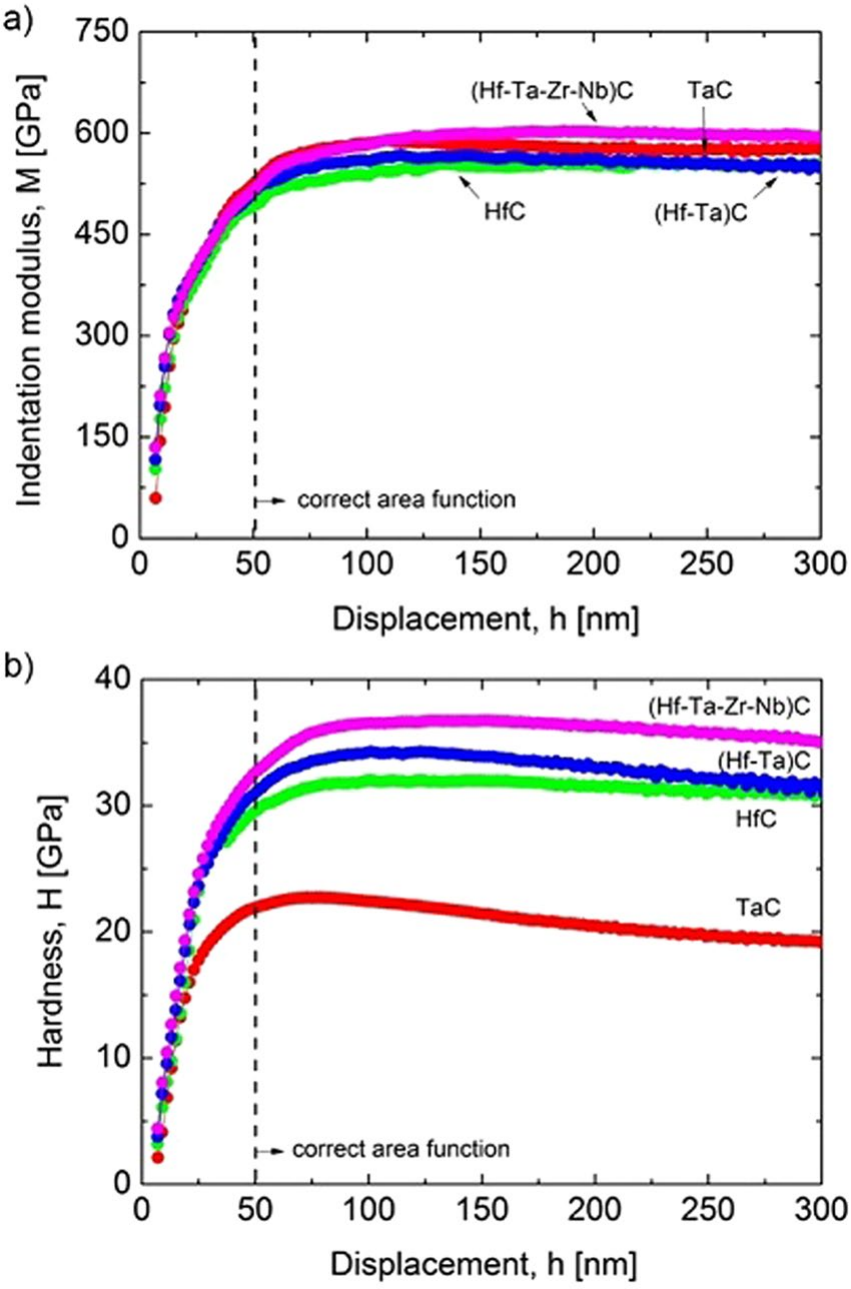
Comparison of results averaged from 64 indents made per sample, following rejection of those close to grain boundaries and pores: (**a**) indentation modulus; and (**b**) hardness depth-profiles of the mono, binary and high-entropy transition metal carbides measured up to 300 nm.

The indentation modulus (*M*) and hardness (*H*) depth-profiles for all of the tested transition metal carbides are plotted in Fig. 4, which are average values taken from the 64 indents which were made per sample, neglecting those which were located near grain boundaries or pores. For clarity, only the materials prepared in the present work are plotted, and without their scatter, which was estimated to be around 2-3% for the modulus and 4-5% for the hardness. This indicates that the indentation measured properties are effectively isotropic. Hardness and Young’s modulus (*E*) depth profiles of ZrC and NbC samples can be found in the publication by Balko *et al*.^33^ (*E* was converted to *M* using Eq. (5) as described in the Methods section).The numerical values for the scatter of the measurements and the averaged *M* and *H* values are given in Table 2. These averages are taken from the region of penetration of 100–300 nm so that the correct area function applies and so that the error introduced by the data from higher depths, which are subject to a more pronounced Indentation Size Effect (ISE, see Methods section), can be minimised. The indentation modulus is practically constant (in the region where the measurement is reliable) showing a moderate increase from ZrC, through HfC~(Hf-Ta)C, and TaC~NbC to the (Hf-Ta-Zr-Nb)C (see Table 2). For the hardness measurements, the depth-profiles show a significant overall increase in hardness from TaC through NbC, ZrC~HfC and (Hf-Ta)C to the (Hf-Ta-Zr-Nb)C together with a slight decrease as a function of depth. This latter effect is known as the indentation size effect (ISE) which is commonly observed in metals and partially in ceramics and is attributed to several factors such as the generation of dislocations, surface effects, cracking, etc.^35^. The hardness and indentation modulus values of TaC and HfC exhibit opposite trends. The TaC grains are stiffer than HfC, while at the same time possess considerably lower hardness than HfC. Similarly, an opposite behaviour is found for ZrC and NbC^33^. This is clear evidence that the hardness is controlled not only by the strength of the atomic bonds, since they exhibit similar elastic properties, but also the number and/or arrangement of active slip systems and strengthening mechanisms. The different deformation behaviour of TaC and HfC grains is attributed to the number of active slip systems, which is twelve for TaC (〈1$\overline{1}$0〉{111} type) and only six for HfC (〈1$\overline{1}$0〉{110} type)^36,37^. Additionally, it has been shown that TaC may also slip on 〈1$\overline{1}$0〉{110}^38^. This is why TaC can undergo a much larger degree of plastic deformation and possess a lower hardness than HfC while exhibiting nearly the same indentation modulus. Additionally, the larger dislocation density in deformed TaC, as reported by De Leon *et al*.^37^, is the reason for its more pronounced ISE in comparison with HfC.

**Table 2:** Comparison of experimental indentation modulus (*M*) and hardness (*H*) values for the investigated mono, binary and high-entropy transition metal carbides.

Material	*M*_*exp*_ [GPa]	*H*_*exp*_ [GPa]
HfC*	552 ± 15	31.5 ± 1.3
TaC*	579 ± 20	20.6 ± 1.2
ZrC^a^	507 ± 16	31.3 ± 1.4
NbC^a^	585 ± 23	27.2 ± 1.7
(Hf-Ta)C*	559 ± 18	32.9 ± 1.8
(Hf-Ta-Zr-Nb)C*	598 ± 15	36.1 ± 1.6
Rule of mixture (HfC, TaC, ZrC, NbC)	556	27.7

^a^Balko *et al*.^33^, *measured in present work.

### Enhanced hardness in binary and high-entropy carbides

The prepared binary and high-entropy transition metal carbides exhibit an enhanced indentation hardness compared to the base monocarbide materials (Table 2). The enhancement in the high entropy carbide is evaluated by comparing the measured indentation hardness value with the rule of mixtures value calculated from the measured values of the component monocarbides. This comparison suggests a significant enhancement in hardness of ~30% (the rule of mixtures calculated indentation hardness value is 27.7 GPa, while the measured value is 36.1 ± 1.6 GPa). However, without knowing the dominant room temperature slip system of the high entropy carbide (the Group IV monocarbides are reported to slip 〈1$\overline{1}$0〉{110} and the Group V monocarbides are reported to slip on 〈1$\overline{1}$0〉{111}, among others)^36–42^ it is important to stress that a direct comparison between the measured hardness and the rule of mixtures calculated hardness is not quantitatively valid. However, it is true to say that the hardness values of the binary (Hf-Ta)C system and the high entropy carbide are significantly higher than even the hardest constituent monocarbide (HfC), and thus there is a real and significant enhancement in the hardness of these materials in comparison to the component monocarbides; raising interesting questions as to the origin of this hardness behaviour.

Recent work by Smith *et al*.^43^ into the dominant slip systems of HfC, TaC and a series of (Hf-Ta)C solid solutions show that even small amounts of Ta added into HfC (with dominant 〈1$\overline{1}$0〉{110} slip) can enable slip on the {111} planes, which they attribute to the introduction of an intrinsic stacking fault on the {111} planes. In this case we might also expect our binary (Hf-Ta)C and the high entropy carbide to slip on the {111} planes. In comparison to the HfC and ZrC, which slip primarily on the {110} planes, this would suggest that these materials may therefore be expected to behave more plastically, with a reduced hardness; due to the greater number of active slip systems available and the fulfilment of the von Mises plasticity criterion. This is contrary to what we have observed. However, as we highlighted in the previous section and as Smith *et al*.^43^ point out, it is not just the availability of slip systems that governs the hardness behaviour, it is also the ease by which lattice slip can occur. This is dependent upon the elastic constants and hence the enhanced hardness may be derived from some interesting bonding behaviour.

Another possibility is that solid solution hardening may be playing some role in the hardness behaviour. Solid solution hardening is observed in metals consisting of different atoms with similar atomic radius and crystal structure. In the case of the high entropy carbide, it is reasonable to suggest that the incorporation of a number of different metallic elements into a single phase solid solution causes local non-uniformities in the lattice structure that further impede the motion of dislocations to produce a solid solution hardening effect. It should be noted that solid solution hardening is a short range interaction, and as a consequence is a thermally activated process. We can therefore anticipate that if solid solution strengthening is significant in high entropy carbides their hardness/yield stress will show a strong dependence on strain rate and temperature.

## Conclusions

We have successfully fabricated a high purity, high density (99%) chemically homogeneous, high entropy carbide ceramic. The work therefore opens up a vast new range of compositional space for the exploration of new UHTC carbides. During the optimisation of the processing route, several observations were made which provide a first insight into the potential behaviour and properties of the (Hf-Ta-Zr-Nb)C material. Firstly, it appears that the lattice parameter mismatch of the component monocarbides may serve as a key indicator for the solid solution formation ability of the high entropy composition; analogous to the use of the Hume-Rothery-based atomic size misfit parameter, *ρ*, in the prediction of solid solution formability in HEAs^3^. Secondly, the observation of chemical decomposition in one of the samples indicates the existence of a miscibility gap in the system. While this indicates that the composition is not thermodynamically stable down to room temperature, it is useful information for the successful fabrication of high entropy carbides and also indicates yet further potential for these compositions to be used to produce *in situ* formed nanocomposites with enhanced mechanical properties. The formation of this decomposed structure in the centre of the sample may also indicate that the high entropy carbide may have a significantly reduced thermal conductivity. Nanoindentation investigations revealed a significant enhancement in hardness (~30%) of the high entropy (Hf-Ta-Zr-Nb)C material compared to that calculated according to the rule of mixtures from the base monocarbides (HfC, TaC, ZrC, NbC) and in comparison to the hardest monocarbide (HfC) and the binary (Hf-Ta)C, which also exhibits a relative hardness enhancement. The mechanism behind this enhancement in hardness may lie in some interesting bonding behaviour leading to enhanced elastic constants. Alternatively, or additionally, some solid solution hardening effects arising from localised lattice strains may be contributing to this increased hardness behaviour. We are undertaking ongoing future work in order to study this interesting behaviour.

## Methods

### Precursor selection and characterisation

In order to find suitably pure starting materials, several of the UHTC monocarbide powders were purchased from both American Elements and H.C. Starck and were compositionally analysed using X-Ray Fluorescence Spectroscopy (XRF, manufacturer – Rigaku, Model - ZSX Primus II) and Light Element Combustion (LECO, manufacturer – LECO, model - ON736 for oxygen and nitrogen, and model - CS230 for carbon) analysis by Kennametal®. The results of the compositional analysis are given in Supplementary Table S1. Based on this analysis, the purest powders (HfC, TaC and TiC from H.C.Starck and ZrC and NbC from American Elements) were selected to make the two target compositions: (Hf-Ta-Zr-Ti)C and (Hf-Ta-Zr-Nb)C.

All of the selected starting carbide powders had a nominal particle size (defined by the supplier) of <44 µm or less; apart from the American Elements ZrC powder which had a stated particle size of <150 µm. For the purpose of encouraging homogeneous inter-diffusion between all of the starting elements, this therefore presented a problem, since the diffusion lengths associated with the ZrC powder would be several times that of the other starting powders. However, on closer inspection of the ZrC powder structure (Supplementary Fig. S6) it was observed that the powder particles actually consist of loosely packed sub-agglomerates. These were broken down during the ball milling process to produce a powder mixture that consisted of powder particles within the same 2–6 µm particle diameter range (Supplementary Fig. S7). Hence, the starting size of the ZrC powder particles was not a concern and could be ruled out when analysing any observed differences in diffusion behaviour.

### Ball milling

To make the selected compositions, the carbide powders were weighed in equiatomic proportions, taking into account the relatively large amount of Hf in the ZrC powder, and mixed by ball milling at 200 rpm for 24 hr (5 min milling, 5 min pause) in WC pots; using SiC milling media, stearic acid as a process control agent and a ball-to-powder ratio of 5:1. The ball milling procedure was designed to mix the powders, rather than to mechanically alloy them, in order to minimise the introduction of impurities such as W from the milling pots and media. All powder handling and ball milling was performed in an argon atmosphere.

### Spark Plasma Sintering (SPS)

The powder mixtures were sintered by Spark Plasma Sintering (SPS) (FCT HPD 25; FCT Systeme GmbH, Rauenstein, Germany). The SPS sintering technique employs a pulsed direct current to provide a rapid Joule heating of the electrically conductive die, tooling and sample (if electrically conductive). This rapid heating in combination with high uniaxial pressures enables materials to be sintered to full density in a matter of minutes as opposed to hours in conventional sintering^44^. For SPS processing, 8 g of each powder mixture was loaded into a 20 mm diameter graphite die, lined with graphite foil. This was then placed into the furnace which was pumped down to a vacuum of around 5 Pa. A pyrometer for process temperature regulation was focussed onto the inside of the top punch of the die, with a 4 mm separation between the point of temperature measurement and the top of the sample; hence the process temperature was not the true temperature of the sample but a nominal temperature. The initial samples were produced following a sintering and pressure profile that was optimised in previous work on the (Hf-Ta)C system^12^, which employed a two-step heating profile with a 10 min dwell at 1800 °C and a 2 min dwell at 2300 °C. The pressure was increased from the minimum 16 MPa to 40 MPa during the dwell at 1800 °C, and was reduced back down to 16 MPa during the 2300 °C dwell. During sintering, heating and cooling rates of 100 °C min^−1^ were employed and a continuous stream of Ar was introduced into the furnace chamber (with the vacuum pump running) to maintain a pressure of around 1200 Pa throughout the process. This reduced the degradation of the graphite die at high temperatures.

Samples of HfC, TaC, and a (Hf-Ta)C with a 1:1 Hf:Ta ratio, were also fabricated using the same selected starting powders, ball milling procedure and SPS sintering program. Direct comparisons could therefore be made between the high entropy carbide and the mono and binary carbides.

### Density, homogeneity and purity characterisation

Each of the starting powders was first characterised by X-ray Diffraction (XRD) (Panalytical X’Pert Pro diffractometer, Cu-kα source, reflection mode, divergence slit, Ni-filter, 5–120 °2θ scanning range, step size 0.0330 °2θ) to check their single-phase nature and to establish the correct reference XRD pattern to check against the final sintered materials. The ball milled mixtures and sintered samples were also characterised using this XRD method.

Both the ball milled powders and sintered samples were investigated by Scanning Electron Microscopy (SEM) and Energy Dispersive X-ray Spectroscopy (EDS) (FEI Inspect™-F field emission gun scanning electron microscope (FEG-SEM)). For EDS analysis, the samples were polished using standard metallographic preparation. Grain pull-out was observed to occur during polishing, resulting in porous-looking surfaces which do not reflect the true density of the materials.

The density of the sintered samples was measured using Archimedes method in water. The theoretical density of the mixed carbide materials was either estimated using a rule of mixtures approximation or, where a single phase solid solution had formed, the XRD-determined lattice parameter was used to calculate the theoretical density based on the assumption of a random distribution of metal atoms on the metallic sublattice.

### Nanoindentation method

The mechanical properties of the prepared TaC, HfC, (Hf-Ta)C and optimised high entropy carbide ((Hf-Ta-Zr-Nb)C) samples were tested using an Agilent/Keysight G200 Nanoindenter equipped with a diamond Berkovich tip. Indents were arranged in arrays of 8 × 8 (i.e. 64 indentations per sample) using a Continuous Stiffness Measuring (CSM) mode with a maximum penetration depth of 300 nm and a distance between the indents of 6 μm. The indents were positioned sufficiently far from each other to neglect the influence of the residual stress field of adjacent indents and to incorporate measurements across several grains of different orientation. To minimise inconsistencies in measurements due to the interaction of the residual stress field of the indents with grain boundaries, pores etc… it was verified that the grain size of the materials was far larger (~20–50 µm) than the size of the indents (~2 µm); and, after testing, indents were analysed by scanning electron microscopy (Zeiss - Auriga) to filter out those that were located at grain boundaries or close to any inclusions (e.g. pores) and to study their morphology. The arrangement of the Berkovich indents are illustrated in the examples for the TaC and (Hf-Ta-Zr-Nb)C samples shown in Supplementary Fig. S5; similar characteristics were observed in the HfC and (Hf-Ta)C samples.

The CSM mode allowed the simultaneous registration of depth, load and stiffness during each test from which the hardness (H) and Young’s modulus (E) depth-profiles were automatically calculated according to the Oliver-Pharr method assuming the individual grains are elastically isotropic^34^. Considering that the tested grains of materials are not perfectly isotropic, instead of the Young’s modulus the indentation modulus (*M*) was derived from the measured stiffness (*S*), via the effective modulus data (*E*_*eff*_), defined in Eqs (1), (2) ^34,45^,

1 $$S=\frac{2\cdot {E}_{eff}}{\sqrt{\pi }}\cdot \sqrt{A}$$

2 $$\frac{1}{{E}_{eff}}=\frac{1}{M}+\frac{1-{\nu }_{i}^{2}}{{E}_{i}}$$

where the first term on the left side of Eq. (2) is related commonly to the Young’s modulus and Poisson’s ratio of the material in isotropic case as follows^34^:

3 $$\frac{1}{M}=\frac{1-{\nu }^{2}}{E}$$

In Eq. (2), the diamond indenter is considered to be isotropic with the Poisson’s ratio of *ν*_i_ = 0.07 and Young’s modulus of *E*_i_ = 1140 GPa. The frame stiffness and tip area functions were calibrated up to 1000 nm prior to the measurements, using a fused silica reference sample which resulted in a correct area function for measurements greater than a ~50 nm depth. The measurements were performed at a constant strain rate of 0.05 s^−1^ and the amplitude and the excitation frequency of the tip were 2 nm and 45 Hz, respectively.

Micromechanical properties of ZrC and NbC were not measured in this particular study but were reported in recent work by the authors^33^. The preparation of the samples was similar to that performed in the present work (powders from H.C. Starck, similar SPS process). Nanoindentation was carried out using the same Agilent G200 machine following identical method on arrays of indents of 10 × 10. Therefore, it is considered that any difference in mechanical behaviour of all four monocarbides (HfC, TaC, ZrC, NbC) would derive only from their intrinsically different physical properties.

### Data availability

The datasets generated during and/or analysed during the current study are available from the corresponding author on reasonable request.

## Electronic supplementary material

Supplementary Information
