# Supplementary material for: Processing and Properties of High-Entropy Ultra-High Temperature Carbides
Source: Sci Rep. 2018 Jun 5;8:8609. doi: 10.1038/s41598-018-26827-1 (PMC5988827; doi:10.1038/s41598-018-26827-1)
Supplement: Supplementary file 1 — Supplementary Information [file 41598_2018_26827_MOESM1_ESM.pdf]

Processing and Properties of High-Entropy Ultra-High Temperature Carbides

Elinor Castle[a*], Tamás Csanádi[b], Salvatore Grasso[a], Jan Dusza[b], Mike Reece[a]

a) Queen Mary, University of London, Mile End Road, London, E1 4NS, UK
b) Institute of Materials Research, Slovak Academy of Sciences, Watsonova 47, 04001, Kosice, Slovakia
*Corresponding author: Room 2.08 Nanoforce, Joseph Priestley Building, Queen Mary, University of London, Mile End Road, London, E1 4NS. UK. E-mail: e.g.castle@qmul.ac.uk

Supplementary Information

Table S1. Compositions of the potential starting Carbide powders as determined by XRF and LECO elemental analysis. The powders which were selected to make the mixed carbide compositions are shown in bold.

Material and supplier	LECO analysed elements (wt.%)			XRF analysed elements (wt.%)										
	C	N	O	Hf	Ta	Zr	Nb	Ti	Cl	Na	W	Al	Fe	Trace elements (<0.1 wt.%)
**HfC (H.C. Starck)**	7.61	0.01	0.54	88.17	0.00	0.26	0.00	2.37	0.76	0.18	0.00	0.01	0.02	Si, S
HfC (American Elements)	5.09	0.62	4.70	78.9	0.00	0.82	0.05	0.00	2.28	0.34	5.45	0.12	0.23	Si, S, Ca, Co, Ni, Ce
**TaC (H.C. Starck)**	7.10	0.01	0.20	0.15	91.17	0.01	0.05	0.22	0.84	0.18	0.00	0.03	0.02	S
ZrC (H.C Starck)	13.88	0.18	0.56	1.77	0.00	79.04	0.00	3.52	0.38	0.10	0.05	0.02	0.03	Si, S, Mn, Ni, Lu, Bi, U
**ZrC (American Elements)**	12.90	0.05	0.20	6.10	0.00	76.42	0.00	0.10	3.63	0.39	0.00	0.15	0.02	Si
**NbC (American Elements)**	12.40	0.11	0.52	0.00	0.13	0.00	83.55	0.69	1.49	0.28	0.46	0.08	0.21	Si, Ca, Re
TiC (American Elements)	20.60	0.32	0.95	0.00	0.00	0.03	0.08	75.28	0.63	0.41	1.12	0.21	0.21	Na, Si, P, S, Cr, Co, Ni,
**TiC (H.C.Starck)**	23.8	0.01	0.27	0.00	0.00	0.00	0.00	75.28	0.20	0.14	0.06	0.01	0.09	Si, S

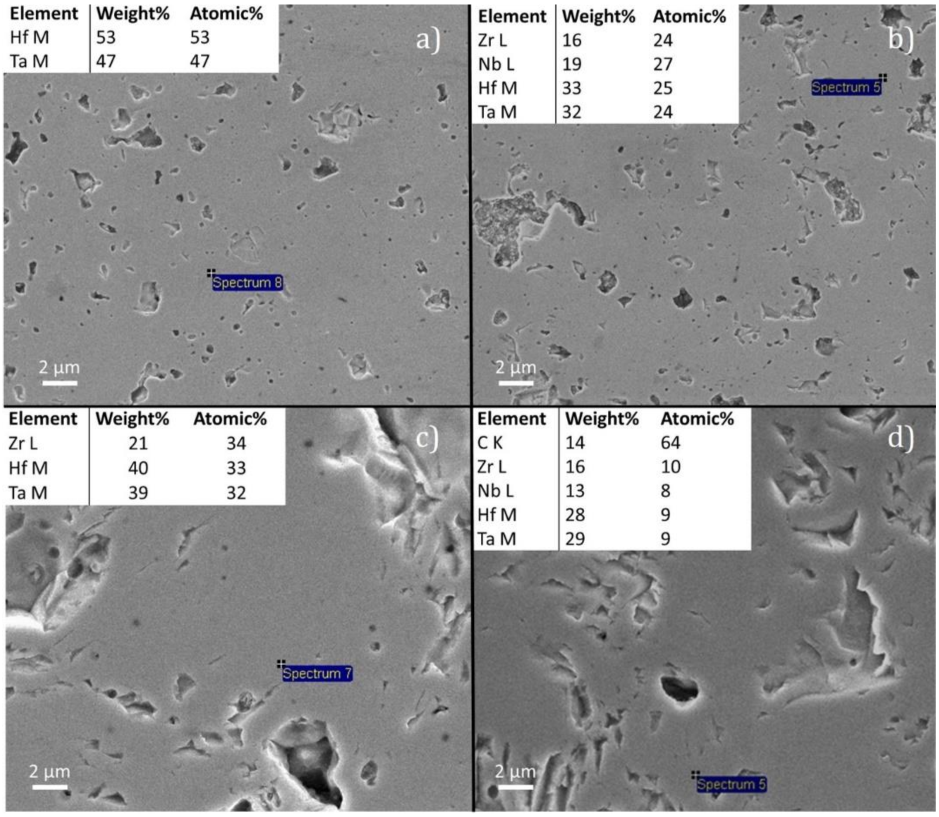

Figure S1. SEM images and EDS analysis of: a) and b) the (Hf-Ta-Zr-Nb)C sample sintered for 2 minutes at 2300 °C and; c) and d) the (Hf-Ta-Zr-Nb)C sample sintered for 5 minutes at 2300 °C, showing the compositional inhomogeneities in the samples.

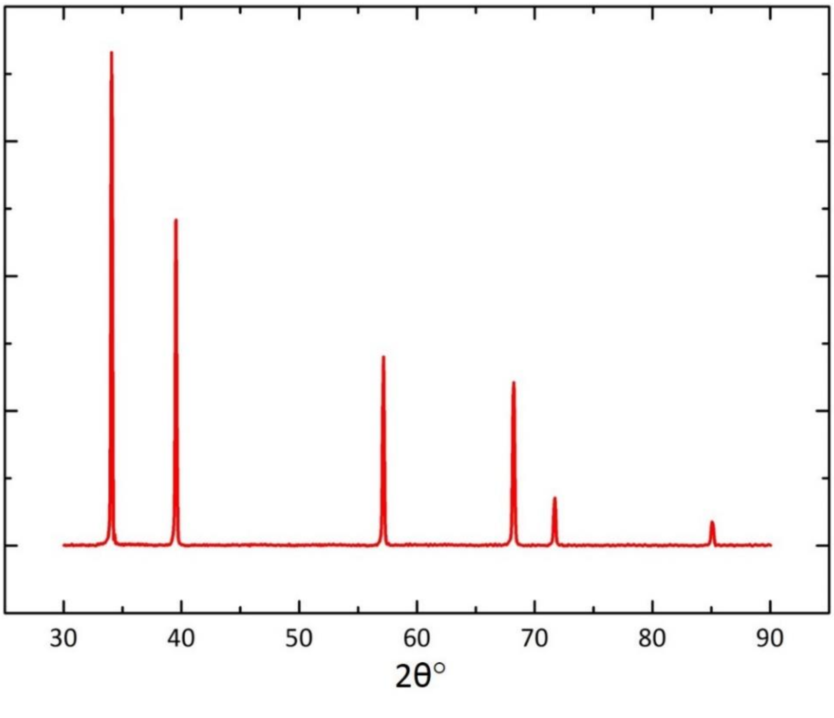

Figure S2. XRD pattern taken from the centre of the (Hf-Ta-Zr-Nb)C sample sintered for 10 min at 2300 °C (the decomposed region) showing what looks like a single phase, indicating that the two regions of the chemical separation have the same crystal structure but perhaps only a slightly different lattice parameter.

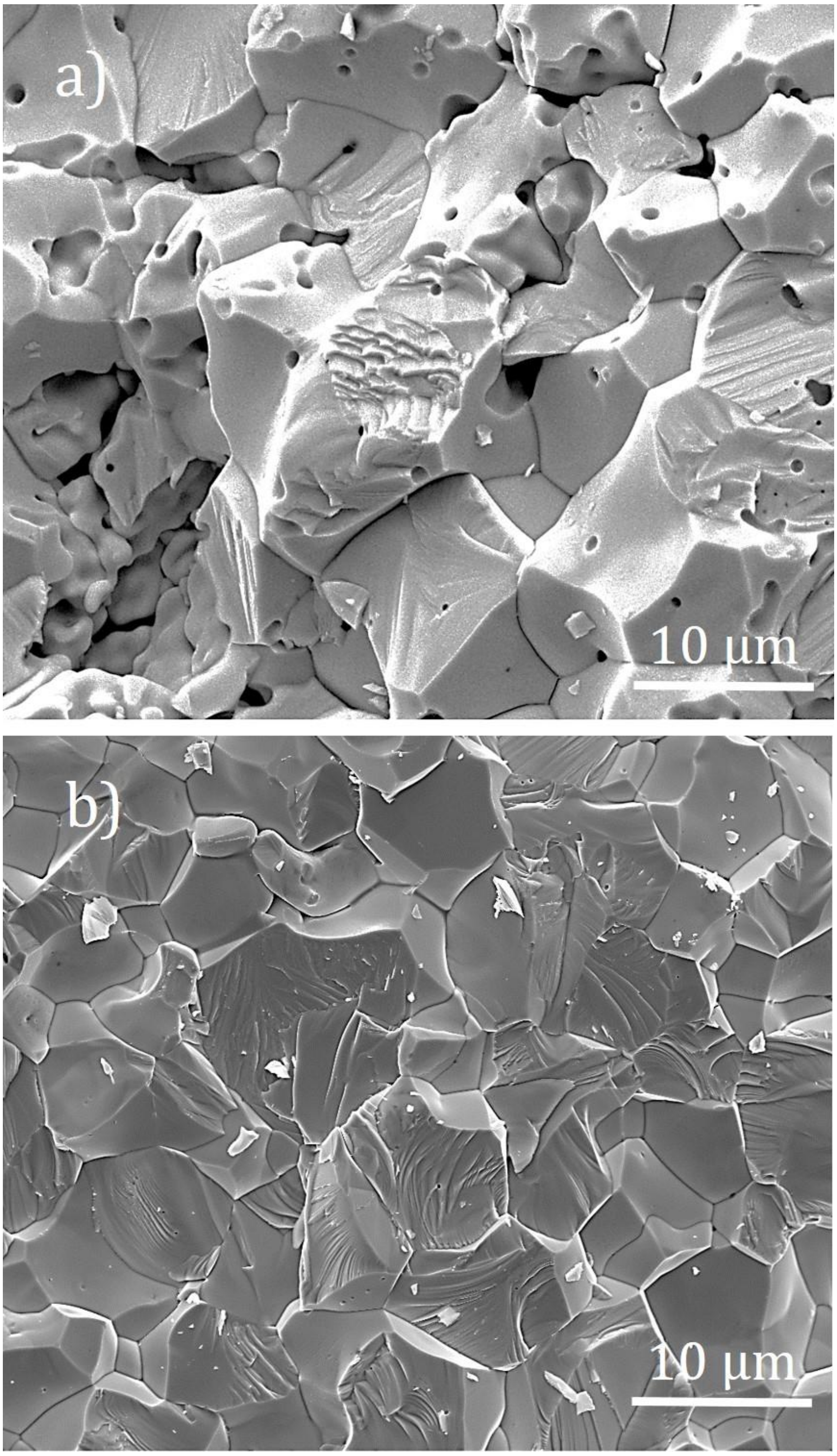

Figure S3. SEM images of the fracture surfaces from: a) a (Hf-Ta-Zr-Nb)C sample which has been processed using the ‘optimised’ processing procedure without an initial pressureless degassing step, showing the spherical pores present in the microstructure due to trapped gases (density 98 %) and; b) a (Hf-Ta-Zr-Nb)C sample which has been processed using pressureless degassing followed by the ‘optimised’ SPS sintering procedure; showing that the pressureless degassing has removed the trapped gas pores from the final microstructure (density 99 %).

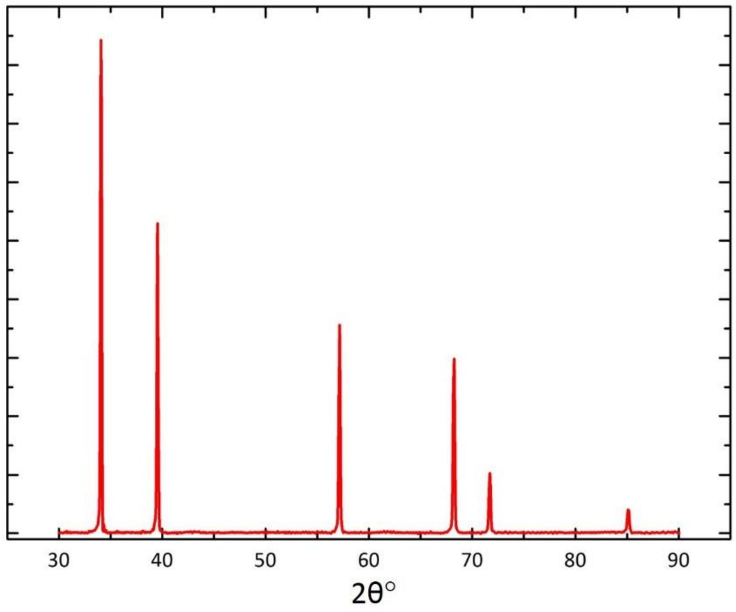

Figure S4. XRD pattern of the ‘optimised’ sample produced by degassing and SPS sintering using a 7 min dwell at 2300 °C. A single phase material is observed with no oxide peaks indicating that a high purity has been achieved.

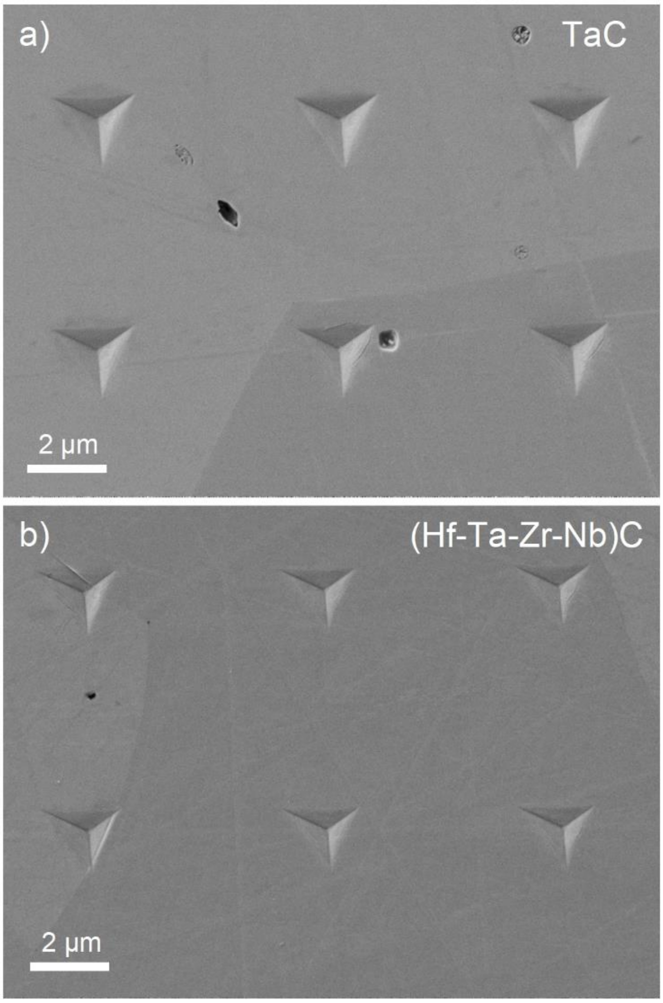

Figure S5. The arrangement of Berkovich indents prepared on: a) TaC; and b) (Hf-Ta-Zr-Nb)C samples.

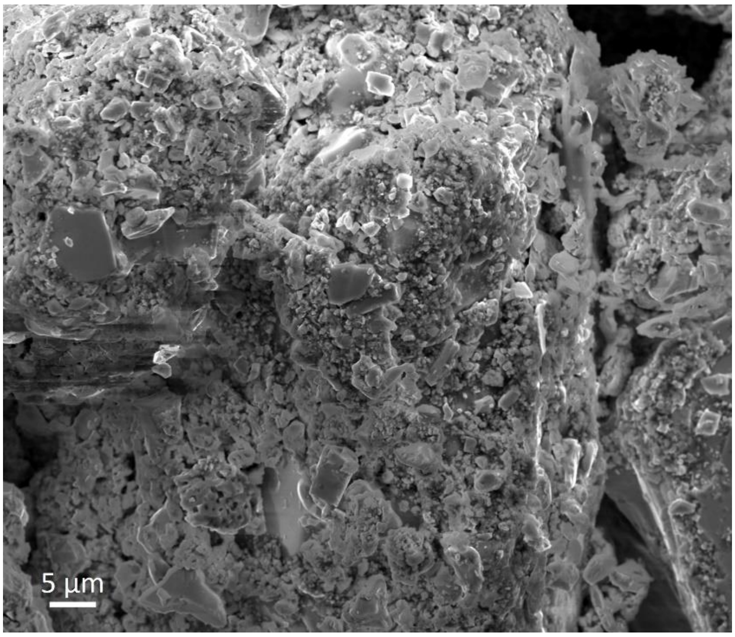

Figure S6. SEM secondary electron image of the selected starting ZrC powder showing that the large powder particles were actually agglomerates of smaller particles which were easily broken down during the ball milling step (see **Figure C**).

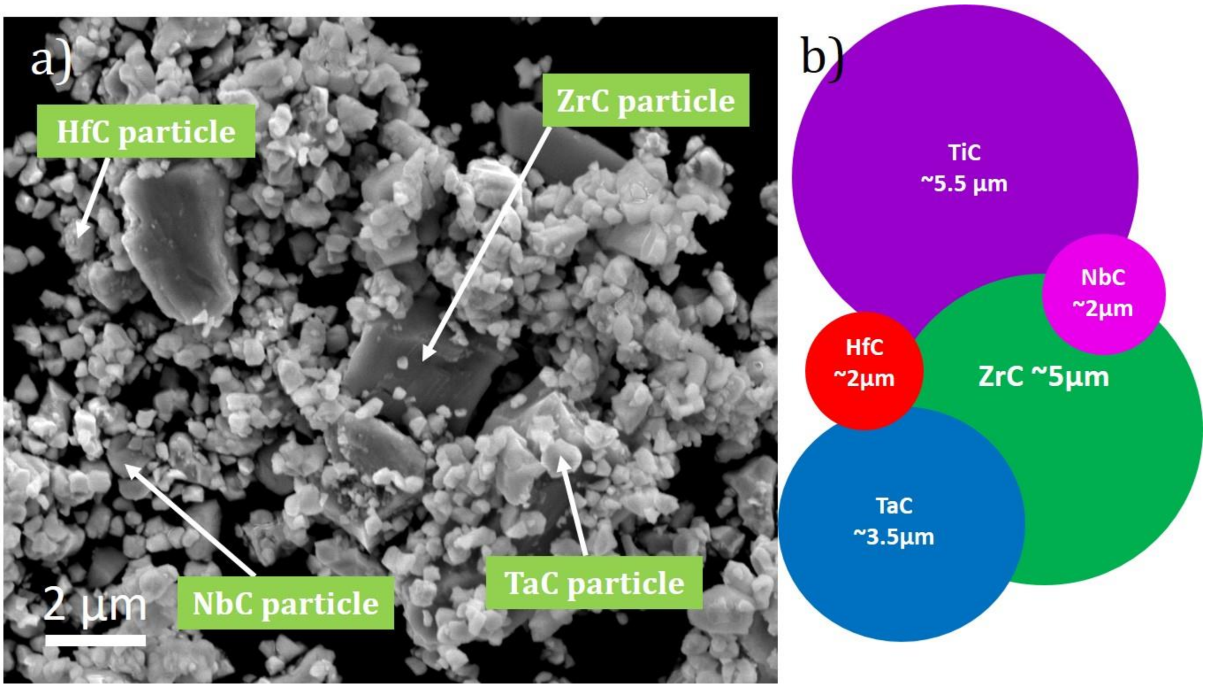

Figure S7. Showing: a) an SEM image of the mixed HfC/TaC/ZrC/NbC powder after ball milling in which it can be seen that the original ZrC agglomerates were broken down; and b) schematic representation of relative average particle sizes in the ball milled mixtures showing a similar particle size and therefore similar relative diffusion distance for each of the elements.
